# Low Prevalence of Human Pathogens on Fresh Produce on Farms and in Packing Facilities: A Systematic Review

**DOI:** 10.3389/fpubh.2018.00040

**Published:** 2018-02-23

**Authors:** Amelia E. Van Pelt, Beatriz Quiñones, Hannah L. Lofgren, Faith E. Bartz, Kira L. Newman, Juan S. Leon

**Affiliations:** ^1^Hubert Department of Global Health, Emory University, Atlanta, GA, United States; ^2^Produce Safety and Microbiology Unit, USDA/ARS/Western Regional Research Center, Albany, CA, United States

**Keywords:** farm-to-fork, pathogen, fruit, vegetable, herb, farm, packing facility

## Abstract

Foodborne illness burdens individuals around the world and may be caused by consuming fresh produce contaminated with bacterial, parasite, and viral pathogens. Pathogen contamination on produce may originate at the farm and packing facility. This research aimed to determine the prevalence of human pathogens (bacteria, parasites, and viruses) on fresh produce (fruits, herbs, and vegetables) on farms and in packing facilities worldwide through a systematic review of 38 peer-reviewed articles. The median and range of the prevalence was calculated, and Kruskal–Wallis tests and logistic regression were performed to compare prevalence among pooled samples of produce groups, pathogen types, and sampling locations. Results indicated a low median percentage of fresh produce contaminated with pathogens (0%). Both viruses (*p*-value = 0.017) and parasites (*p*-value = 0.033), on fresh produce, exhibited higher prevalence than bacteria. No significant differences between fresh produce types or between farm and packing facility were observed. These results may help to better quantify produce contamination in the production environment and inform strategies to prevent future foodborne illness.

## Introduction

The World Health Organization reported a global burden of 600 million cases of foodborne illness and 420,000 foodborne illness-attributed deaths in 2010 (1). The majority of foodborne illnesses result from viral pathogens, such as norovirus, succeeded by bacterial pathogens, such as *Salmonella*, and parasite pathogens, such as *Cyclospora* (1, 2). Foodborne illness may be caused by contaminated fresh produce [reviewed in Ref. (3–5)]. For example, from 2004 to 2013 in the United States, 36% of cases of foodborne illnesses resulted from consumption of contaminated produce (6).

Fresh produce can become contaminated with human pathogens during harvesting on farms and packaging either in the field or in packing facilities (7, 8). For example, investigations into an outbreak of *Salmonella* in the United States in 2008 identified jalapeño farms in Mexico as the source of contamination (9, 10). Further, investigators of a norovirus outbreak in Denmark in 2010 concluded that lettuce from a producer in France caused the infection (11). In a third example, researchers traced the source of a multistate outbreak of *Listeria monocytogenes* in the United States in 2011 to contamination of cantaloupe in packing facilities in Colorado (12). Thus, produce may become contaminated in one region but affect consumers in other regions.

Although outbreak data provides information on common pathogens and produce types involved in foodborne illness of the consumer, it often does not identify or quantify the source of contamination in the original setting at the farm or packing facility [reviewed in Ref. (3–5)]. This article aims to determine the prevalence of human pathogens (bacteria, parasites, and viruses) on fresh produce (herbs, fruits, and vegetables) on farms and in packing facilities worldwide through a systematic analysis of peer-reviewed literature published before August 2017.

## Methods

### Article Identification

A search for peer-reviewed literature published in English or Spanish from the initial date of each database to July 2017 was performed in four databases: PubMed, Web of Science, Academic Search Complete, and Agricola. Variables were compiled to create a search string for produce type, farm or packing facility, prevalence data, pathogen type, and excluding common non-produce routes of contamination (Table 1). The initial search completed on November 2015 yielded 840 articles across all databases. An additional identical search completed on July 2017 yielded 127 new articles for a total of 967 articles across all databases.

**Table 1:** Overview of search string operations.

Database	Search string	Search field	Article yield
Academic search complete	(vegetable* OR fruit*) AND (farm* OR packing OR packag* OR process*) AND (prevalence OR contamination) AND (*Escherichia coli* OR hepatitis OR salmonella OR norovirus OR botulinum OR pathogen* OR bacteria OR virus*) NOT (meat OR dairy OR fish)	Abstract	240
Agricola	(vegetable* OR fruit*) AND (farm* OR packing OR packag* OR process*) AND (prevalence OR contamination) AND (*Escherichia coli* OR hepatitis OR salmonella OR norovirus OR botulinum OR pathogen* OR bacteria OR virus*) NOT (meat OR dairy OR fish)	Abstract	130
PubMed	((vegetable OR vegetables OR fruit OR fruits) AND (farm OR farms OR packing OR packag* OR process*) AND prevalence AND (*Escherichia coli* OR hepatitis OR salmonella OR norovirus OR botulinum OR contaminat* OR pathogen* OR bacteria OR virus*) NOT (meat OR dairy OR fish))	All fields	348
Web of science	((vegetable$ OR fruit$) AND (farm$ OR packing OR packag* OR process*) AND prevalence AND (*Escherichia coli* OR hepatitis OR salmonella OR norovirus OR botulinum OR contaminat* OR pathogen$ OR bacteria OR virus*) NOT (meat OR dairy OR fish))	Topic	249

### Article Screening

After removing duplicate articles across databases, the remaining 706 articles were subjected to inclusion and exclusion criteria by two independent reviewers (Amelia E. Van Pelt and Hannah L. Lofgren), and discrepancies were reconciled through discussions or a third independent reviewer (Juan S. Leon). Inclusion criteria included articles with: (1) English or Spanish language, (2) produce samples tested that came from a farm or packing facility, (3) fresh produce samples that were not processed (e.g., not frozen, peeled, cut, or rinsed with disinfectant), (4) reported prevalence (percent or whole number) of human foodborne pathogens, and (5) produce samples that were individual (e.g., not composite). Exclusion criteria excluded articles that reported outbreaks, lab-based studies, non-human pathogens indicator organisms, insufficient information on whether it was sampled from a farm or packing facility or prevalence data, microorganisms not strongly associated with human (e.g., *Enterobacter* or *Enterococcus*), or non-foodborne pathogens (e.g., *Raoultella terrigena, Tatumella terra, Pantoea agglomerans, Pseudomonads, Rahnella aquatilis*, and *Serratia fonticola*). Application of inclusion and exclusion criteria resulted in 38 articles.

### Data Extraction

From each of the selected 38 articles, data were extracted independently by two reviewers (Amelia E. Van Pelt and Hannah L. Lofgren), and discrepancies were documented and reconciled through discussion or a third independent reviewer (Juan S. Leon). Extracted data included: article title, first author, journal, publication date, pathogen(s) examined, produce examined, study city/state/region, study country, study contamination location, month/season of produce collection, laboratory detection method(s), and statistical measurement unit (e.g., percent or CFU). Further, in the article, if the same individual produce commodities were sampled from the same location and analyzed at one time using the same laboratory methodology, then they were defined as a group. For example, two separate farm visits where 12 cantaloupes per visit were sampled from the same farm location and analyzed at one time using the same laboratory methodology were considered two groups of cantaloupes for that article. From each article, the following data were extracted: the number of positive pathogen observations of each group (prevalence numerator), number of individual commodities tested at one time (prevalence denominator) of each group, and the group pathogen prevalence (prevalence numerator divided by prevalence denominator). When only numerator and denominator data were reported, reviewers manually calculated the pathogen prevalence.

### Statistical Analysis

Descriptive analyses were performed using SAS version 9.4 (SAS Institute, Cary, NC, USA). The prevalence of human pathogens (bacteria, parasites, and viruses) on fresh fruits, herbs, and vegetables was estimated across groups. To account for the non-normal distribution and small sample size of data, the median and range across groups were calculated to more accurately measure the central tendency of the prevalence percentage. In addition, the sample location of the commodities tested was inferred based on the authors’ description in the methods and categorized into farm and packing facility for analysis, and analyses were stratified by farm and packing facility. Commodities of the same genus were grouped together for analysis, but the Supplementary Materials include all commodities as separate species. Kruskal–Wallis tests and Steel Dwass tests, where appropriate, were completed to compare the continuous prevalence across pathogens, commodities, and farm vs packing facility. To compare the dichotomous prevalence (0% prevalence vs >0% prevalence), logistic regression was performed. All groups were pooled for respective analyses, with the limitation that pooling data extracted from individual articles across regions, laboratory methods, and time may have widened the error and variability of the resulting estimates.

## Results

**Figure 1:**
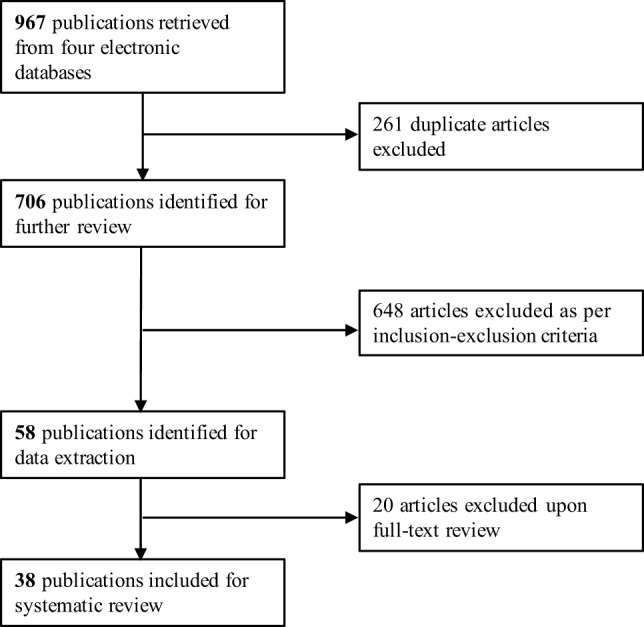
Flow chart of literature selection for systematic review. The vertical arrows indicate inclusion, and the horizontal arrows indicate exclusion.

The initial search yielded 967 articles [PubMed (348 articles), Web of Science (249), Academic Search Complete (240), and Agricola (130)] (Figure 1). After removing 261 duplicate articles, 706 remained for further review. 648 articles were excluded as per criteria (see “Materials and Methods”); many studies occurred in markets and produce stands instead of farms and packing facilities. Through the data extraction process of 58 articles, 20 additional articles were excluded, because they did not contain sufficient information on pathogen prevalence, whether it was sampled from a farm or packing facility, or produce type to enable accurate analysis; most publications did not stratify their prevalence data by produce type. The remaining 38 articles contributed data to this review. Thirty-five articles sampled from farms, and eight articles sampled from packing facilities. Ten articles sampled from North America, 10 articles sampled from Asia, 9 articles sampled from Europe, and 7 articles sampled from Africa. Only one article sampled from South America. Among pathogens tested, *Salmonella* was tested the most (61%, *n* = 23 articles) and among commodities tested, lettuce was sampled the most (37%, *n* = 14 articles).

### Pathogen Type

To determine the prevalence of human pathogens on fresh produce, researchers extracted, or manually calculated, prevalence data from each article (see “Materials and Methods”). The median and range of the percent of positive samples across produce groups (see “Materials and Methods” for definition of a produce group) from farms and packing facilities were calculated (Tables 2 and 3). The majority of articles tested for bacteria, (84%) followed by parasites (18%) and viruses (5%). Across farms and packing facilities combined (Tables 2 and 3), among bacteria, *Campylobacter* had the highest median prevalence (25%, *n* = 18), while pathogenic *Escherichia coli* (referred to as enterohemorrhagic *E. coli* (EHEC), Shiga toxin/verotoxin-producing *E. coli* (STEC/VTEC), or *E. coli* O26, O103, O111, O145, and O157:H7), *Listeria*, and *Salmonella* had the lowest median prevalence (0%, *n* = EHEC: 1, STEC/VTEC: 13, O26-O145:4, O157H7: 51, *Listeria*: 45, and *Salmonella*: 84). Among parasites, *Giardia* had the highest median prevalence (50%, *n* = 13), and *Ascaris* spp., *Trichuris* spp., and *Toxocara* spp. had the lowest median prevalence (0%, *n* = 40). Among viruses, norovirus had the highest median prevalence of positive samples (22%, *n* = 9), while rotavirus had the lowest median prevalence (0%, *n* = 6). When pooling and comparing bacteria, parasites, and viruses to each other, the prevalence of viruses (Steel Dwass = 3.869, *p*-value = 0.017) and parasites (Steel Dwass = 3.541, *p*-value = 0.033) on fresh produce were both significantly higher than the prevalence of bacteria. When produce groups were reclassified to presence vs absence of pathogens, there was no significant difference in the percentage of groups that had pathogens among viruses, parasite, or bacteria (bacteria was the referent group; virus: OR 3.166, 95% CI 0.813–12.329, *p*-value = 0.154; parasite: OR 1.378, 95% CI 0.879–2.161, *p*-value = 0.527).

**Table 2:** Prevalence of pathogens on produce from farms by pathogen type.

Pathogen	Median prevalence^a^ (range)	Number of groups sampled^b^	Sample country	Reference
**Bacteria**				
*Bacillus cereus*	7.9 (0–11.1)	6	Korea	(13)
*Campylobacter*	25.0 (0–90.0)	18	Belgium, Malaysia	(14, 15)
*Escherichia coli* O157:H7	0 (0–1.6)	51	Brazil, Korea, Multi-country^c^, South Africa, Spain, United States	(13, 16–22)
*E. coli* O26, O103, O111, O145	0	4	Spain	(16)
Enterohemorrhagic *E. coli*	0	1	Belgium	(23)
Enteric *E. coli*^d^	0	13	Greece, Japan, United States	(24–26)
*Hafnia alvei*	10.0 (0–20.0)	2	Netherlands, Poland	(27, 28)
*Listeria*	0 (0–20.0)	43	Germany, India, Korea, Multi-country^c^, Poland, South Africa, Spain, United States	(13, 16–18, 22, 29–33)
*Salmonella*	0 (0–60.0)	80	Belgium, Brazil, China, Egypt, Eritrea, Germany, Japan, Kenya, Korea, Lebanon, Mexico, Multi-country^c^, Poland, South Africa, Spain, United States	(13, 16–21, 23, 24, 26, 28, 30, 33–41)
*Shigella*	0 (0–8.3)	7	Eritrea, United States	(37, 38)
*Staphylococcus aureus*	5.5 (1.0–7.9)	8	China, Korea	(13, 39)

**Parasite**				
*Ascaris* spp.	0 (0–33.3)	40	Poland	(42)
*Cryptosporidium*	8.9 (0–50.0)	13	Cambodia, Iran	(43, 44)
*Cyclospora*	10.4 (0–17.0)	15	Cambodia, Italy, Vietnam	(44–46)
*Giardia*	50.0 (0–100)	13	Cambodia, Eritrea	(38, 44)
Helminth eggs	9.7 (2.80–11.1)	4	Cambodia	(44)
*Toxocara* spp.	0 (0–100)	40	Poland	(42)
*Trichuris* spp.	0 (0–33.3)	40	Poland	(42)

**Virus**				
Norovirus	30.0 (23.3–40.0)	3	Multi-country^c^, Spain	(17)

*^a^Median % of produce samples positive*.

*^b^Number of groups of commodities*.

*^c^Data aggregated for more than one country*.

*^d^Shiga toxin-producing E. coli and Verotoxin-producing E. coli*.

**Table 3:** Pathogen prevalence on produce from packing facilities by pathogen type.

Pathogen	Median prevalence^a^ (range)	Number of groups sampled^b^	Sample country	Reference
**Bacteria**				
*E. coli* O157:H7	5.0 (0–10.0)	2	Multi-country^c^, South Africa	(33, 47)
*Listeria*	4.8 (4.7–4.8)	2	United States	(31)
*Salmonella*	0 (0)	5	South Africa, United States	(33, 40, 48, 49)
*Shigella*	0 (0)	2	Multi-country^c^, United States	(37, 47)
*Staphylococcus aureus*	5.00	1	South Africa	(33)

**Virus**				
Hepatitis A	11.1 (0–50.0)	6	Mexico	(50)
Norovirus	10.0 (0–50.0)	6	Mexico	(50)
Rotavirus	0 (0–11.1)	6	Mexico	(50)

*^a^Median % of produce samples positive*.

*^b^Number of groups of commodities*.

*^c^Data aggregated for more than one country*.

### Farms and Packing Facilities

To contrast the prevalence of human pathogens on fresh produce between farms and packing facilities, researchers stratified the results by farms (Table 2) and packing facilities (Table 3). In this systematic review, the majority of articles (92%) sampled from farms. When stratified by farm, the pathogens with the highest median prevalence, described in the preceding paragraph, did not change. The articles that sampled from packing facilities only tested for bacteria and viruses, not parasites. In contrast to the pathogen prevalence on farms, in packing facilities, *E. coli* O157:H7 had the highest median prevalence among bacteria (5.0%, *n* = 2), and hepatitis A had the highest median prevalence (11.1%, *n* = 6) among all pathogens included. In packing facilities, *Salmonella* and *Shigella* were not detected, and rotavirus was rarely detected. Overall, there was no significant difference in the prevalence of pathogens between farms and packing facilities.

### Produce Type

To compare the prevalence of human pathogens across fresh produce types, researchers stratified the results by herbs (Table 4), fruits (Table 5), and vegetables (Tables 6 and 7). The minority of articles sampled herbs (26%, *n* = 10 articles), followed by fruits (45%, *n* = 17 articles) and vegetables (82%, *n* = 31 articles). Among herbs, Vietnamese coriander (see Supplementary Materials) had the highest median prevalence of samples positive with bacteria (41%, *n* = 2), while marjoram had the highest median prevalence of samples positive with parasites (17%, *n* = 1). Among all herbs, only cilantro (20%, *n* = 3) and parsley (50%, *n* = 3) tested positive for viruses. Among fruits, peaches had the highest median prevalence of samples positive with bacteria (2.5%, *n* = 5), while raspberries had the highest median prevalence of samples positive with viruses (40%, *n* = 1). No parasites were detected on fruit. Among vegetables, wild cosmos had the highest median prevalence of samples positive with bacteria (65%, *n* = 4), while rhubarb had the highest median prevalence of samples positive for parasites (33%, *n* = 6). After pooling and comparing all commodities by herbs, fruits, and vegetables, there was no significant difference in the prevalence of pathogens by produce commodity type.

**Table 4:** Pathogen prevalence by herb commodity.

Commodity	Median prevalence^a^ (range)	Number of groups sampled^b^	Pathogens tested	Sample country	Reference
Basil	6.8 (1.1–12.5)	2	*Cryptosporidium, Cyclospora*	Iran, Vietnam	(43, 45)
Coriander^c^	8.3 (0–70.0)	11	*Campylobacter, Cryptosporidium, Cyclospora, Escherichia coli* O157:H7, Hepatitis A, *Listeria*, norovirus, rotavirus, *Salmonella*	Iran, Malaysia, Mexico, United States, Vietnam	(14, 15, 22, 43, 45, 50)
Cress	8.9	1	*Cryptosporidium*	Iran	(43)
Fennel	0	6	*Ascaris* spp., *Toxocara* spp., *Trichuris* spp.	Poland	(42)
Dill	0	5	*E. coli* O157:H7, *Listeria, Salmonella*, Shiga toxin-producing *E. coli*	Greece, Poland, United States	(22, 25, 28)
Indian pennywort	0	1	*Campylobacter*	Malaysia	(14)
Marjoram	16.6	1	*Cyclospora*	Vietnam	(45)
Mint^d^	8.1 (7.6–8.5)	2	*Cryptosporidium, Cyclospora*	Iran, Vietnam	(43, 45)
Parsley	0 (0–50.0)	14	*Ascaris* spp., *E. coli* O157:H7, Hepatitis A, *Listeria*, norovirus, rotavirus, *Salmonella*, Shiga toxin-producing *E. coli, Toxocara* spp., *Trichuris* spp.	Greece, Lebanon, Mexico, Poland, United States	(22, 25, 34, 42, 50)
Sorrel	0	6	*Ascaris* spp., *Toxocara* spp., *Trichuris* spp.	Poland	(42)

*^a^Median % of produce samples positive*.

*^b^Number of groups of commodities*.

*^c^Cilantro, coriander, and Vietnamese coriander*.

*^d^Mint and Vietnamese mint*.

**Table 5:** Pathogen prevalence by fruit commodity.

Commodity	Median prevalence^a^ (range)	Number of groups sampled^b^	Pathogens tested	Sample country	Reference
Apple	0 (0)	4	*Escherichia coli* O157:H7, *Salmonella*	United States	(21)
Blackberry	0	1	*Listeria*	Poland	(29)
Blueberry	0	1	*Listeria*	Poland	(29)
Cantaloupe	1.7 (0–40.0)	4	*E. coli O157:H7, Listeria, Salmonella*	Mexico, United States	(22, 36)
Citrus^c^	0 (0)	4	*Salmonella*, Verotoxin-producing *E. coli*	Japan, United States	(26, 51)
Fruit^d^	0 (0–4.00)	6	*E. coli* O157:H7, *Listeria, Salmonella*	Germany, South Africa, United States	(19, 30, 40)
Kiwifruit	0.50 (0–3.9)	6	Pathogenic *E. coli, Salmonella, Staphylococcus aureus*	China	(39)
Peach	2.5 (0–10.0)	5	*E. coli* O157:H7, *Listeria, Salmonella, S. aureus*	South Africa	(33)
Raspberry	0 (0–40.0)	5	*E. coli* O157:H7, *Listeria*, norovirus, *Salmonella*	Multi-country^e^, Poland	(17, 29)
Strawberry	0 (0–30.0)	15	*Ascaris* spp., *E. coli* O157:H7, *Listeria*, norovirus, *Salmonella, Toxocara* spp., *Trichuris* spp.	Poland, Spain, United States	(17, 21, 29, 42)

*^a^Median % of produce samples positive*.

*^b^Number of groups of commodities*.

*^c^Orange, Satsuma mandarin, and tangerine*.

*^d^Specific commodities not articulated*.

*^e^Data aggregated for more than one country*.

**Table 6:** Pathogen prevalence by vegetable commodity.

Commodity	Median prevalence^a^ (range)	Number of groups sampled^b^	Pathogens tested	Sample country	Reference
Arugula	0	3	*Escherichia coli* O157:H7, *Listeria, Salmonella*	United States	(22)
Beetroot	16.7 (0–16.7)	8	*Ascaris* spp., Salmonella, Shiga toxin-producing *E. coli, Toxocara* spp., *Trichuris* spp.	Greece, Poland	(25, 28, 42)
Bok choi	0	4	*E. coli* O157:H7, *Salmonell*a	United States	(21)
Brinjal	20.0	1	*Listeria*	India	(32)
Broccoli	0	11	*Ascaris* spp*., E. coli* O157:H7, *Listeria, Salmonella, Toxocara* spp*., Trichuris* spp.	India, Poland, United States	(21, 32, 42)
Bulbous vegetables^c^	0.9 (0–1.7)	2	*Listeria, Salmonella*	Germany	(30)
Cabbage	0 (0–50.0)	25	*Ascaris* spp*., Campylobacter, E. coli* O157:H7, *Giardia*, Hepatitis A, *Listeria*, norovirus, rotavirus, *Salmonella, Shigella, Toxocara* spp*., Trichuris* spp.	Egypt, Eritrea, India, Malaysia, Mexico, Poland, South Africa, United States	(14, 21, 29, 31, 32, 38, 41, 42, 50, 52)
Carrot	0 (0–42.9)	15	*Ascaris* spp*., Giardia, Salmonella*, Shiga toxin-producing *E. coli, Shigella, Toxocara* spp*., Trichuris* spp.	Eritrea, Greece, Poland, United States	(25, 28, 37, 38, 42)
Cauliflower	0 (0–20.0)	7	*Ascaris* spp*., Listeria, Toxocara* spp*., Trichuris* spp.	India, Poland	(32, 42)
Celery	0 (0–25.0)	8	*Ascaris* spp*., Cyclospora*, Shiga toxin-producing *E. coli, Toxocara* spp*., Trichuris* spp.	Greece, Italy, Poland	(25, 42, 46)
Chappan-kaddu	10.0	1	*Listeria*	India	(32)
Chile pepper	60.0	1	*Salmonella*	Mexico	(36)
Chili	10.0	1	*Listeria*	India	(32)
Collards	0	3	*E. coli* O157:H7, *Listeria, Salmonella*	United States	(22)
Cowpea	0	1	*Listeria*	India	(32)
Cucumber	0 (0–70.0)	7	*Campylobacter, Cyclospora, E. coli* O157:H7, *Salmonella*	Italy, Malaysia, United States	(15, 21, 46)
Cucurbits	0	3	*Giardia, Salmonella, Shigella*	Eritrea	(38)
Dolichos bean	20.0	1	*Listeria*	India	(32)
French beans	0	6	*Ascaris* spp., *Toxocara* spp., *Trichuris* spp.	Poland	(42)
Garlic	0	1	Shiga toxin-producing *E. coli*	Greece	(25)
Green onion	18.5 (11.1–22.2)	4	*Cryptosporidium*, Hepatitis A, norovirus, rotavirus	Iran, Mexico	(43, 50)
Green pepper	0 (0–2.3)	4	*E. coli* O157:H7, *Salmonella*	United States	(21)
Jalapeño pepper	0	3	Hepatitis A, norovirus, rotavirus	Mexico	(50)

*^a^Median % of produce samples positive*.

*^b^Number of groups of commodities*.

*^c^Specific commodities not articulated*.

**Table 7:** Pathogen prevalence by vegetable commodity continued.

Commodity	Median prevalence^a^ (range)	Number of groups sampled^b^	Pathogens tested	Sample country	Reference
Kale	0	2	*Salmonella*	Kenya	(35)
Leafy greens	0 (0–25.0)	7	*Escherichia coli* O157:H7, *Giardia, Salmonella, Shigella*	Eritrea, United States	(21, 38)
Leek	0 (0–8.3)	8	*Ascaria* spp*., Cryptosporidium*, Shiga toxin-producing *E. coli, Toxocara* spp*., Trichuris* spp.	Greece, Iran, Poland	(25, 42, 43)
Lettuce^c^	0 (0–50.0)	40	*Bacillus cereus, Campylobacter, Cyclospora, E. coli* O157:H7,*Hafnia alvei*, Hepatitis A, *Listeria*, norovirus, rotavirus, *Salmonella*, Shiga toxin-producing *E. coli, Staphylococcus aureus, Toxocara* spp., *Trichuris* spp.	Belgium, Brazil, Egypt, Eritrea, Greece, Korea, Lebanon, Mexico, Netherlands, Poland, South Africa, United States, Vietnam	(13, 20, 21, 23, 25, 27–29, 34, 38, 41, 42, 45, 50, 52)
Long yard bean	25.0 (0–50.0)	2	*Campylobacter*	Malaysia	(14, 15)
Mustard greens	0	3	*E. coli* O157:H7, *Listeria, Salmonella*	United States	(22)
Onion	0 (0–28.6)	14	*Ascaris* spp., *E.coli* O157:H7, *Listeria, Salmonella, Trichuris* spp., *Toxocara* spp.	Greece, Poland, South Africa, United States	(18, 21, 25, 42)
Palak	0	1	*Listeria*	India	(32)
Potato	0 (0–33.3)	6	*Ascaris* spp., *Toxocara* spp., *Trichuris* spp.	Poland	(42)
Pumpkin	0	6	*Ascaris* spp., *Toxocara* spp., *Trichuris* spp.	Poland	(42)
Radish	0 (0–16.7)	3	*Campylobacter, Salmonella*	Lebanon, Malaysia, Poland	(14, 28, 34)
Rhubarb	33.3 (0–33.3)	6	*Ascaris* spp., *Toxocara* spp., *Trichuris* spp.	Poland	(42)
Root vegetables^f^	1.5 (0–3.0)	2	*Listeria, Salmonella*	Germany	(30)
Salad	0.8 (0–1.6)	2	*Listeria, Salmonella*	Germany	(30)
Sesame leaf	0 (0–7.9)	10	*Bacillus cereus, E.coli* O157:H7, *Listeria, Salmonella, S. aureus*	Korea	(13)
Spinach^d^	5.45 (0–100)	52	*Bacillus cereus, Campylobacter, Cryptosporidium, Cyclospora, E.coli* O157:H7, *Giardia*, Helminth eggs, *Listeria, Salmonella*, Shiga toxin-producing *E.coli, Shigella, S. aureus*	Cambodia, Greece, Korea, Malaysia, Multi-country, South Africa, Spain, United States	(13, 15, 16, 22, 25, 44, 47, 52)
Summer squash	0	4	*E. coli* O157:H7, *Salmonella*	United States	(21)
Tomato^e^	0 (0–23.3)	16	*Campylobacter, E. coli* O157:H7, *Giardia, Listeria*, norovirus, *Salmonella*, Shiga toxin-producing *E. coli, Shigella*	Eritrea, India, Malaysia, Poland, Spain, United States	(14, 17, 21, 24, 29, 32, 38)
Turnip	0	6	*Ascaris* spp., *Toxocara* spp., *Trichuris* spp.	Poland	(42)
Vegetables^f^	0	2	*E. coli* O157:H7, *Salmonella*	United States	(19)
Wild cosmos	65.0 (30.0–90.0)	4	*Campylobacter*	Malaysia	(15)
Winged bean	6.7	1	*Campylobacter*	Malaysia	(15)
Young beetroot leaves	0 (0–100)	6	*Ascaris* spp*., Toxocara* spp*., Trichuris* spp.	Poland	(42)
Zucchini	0 (0–14.3)	10	*Ascaris* spp*., E. coli* O157:H7, *Salmonella, Toxocara* spp*., Trichuris* spp.	Poland, United States	(21, 42)

*^a^Median % of produce samples positive*.

*^b^Number of groups of commodities*.

*^c^Lettuce and Romaine lettuce*.

*^d^Baby spinach, Roman rocket, spinach, and water spinach*.

*^e^Tomato and cherry tomato*.

*^f^Specific commodities not articulated*.

## Discussion

This research aimed to quantify the prevalence of contamination of human pathogens on fresh produce on farms and packing facilities. Overall, pathogens were detected on a low median percentage of fresh produce from farms and packing facilities. This low percentage may result from the environment on the farms and in the packing facilities. Further, the enforcement of produce safety guidelines (e.g., US Good Agricultural Practices and GlobalGAP) may also limit the opportunities for pathogen contamination to occur on farms and in packing facilities (53–56). Specifically, the handling practices of the fresh produce may reduce the opportunities for the transfer of human pathogens to the fresh produce (54, 56). In addition, the environmental conditions such as sunlight, moisture, the physical characteristics of the surface of the produce, and temperature may limit the opportunities for pathogen persistence (57–59). Of note, results indicated a variance in the range of pathogen prevalence. Certain commodities, farms and packing facilities, and pathogens exhibited a wide range of pathogen prevalence (e.g., 0–100), while others had limited range (e.g., 0); however, most still had a median of 0% or low pathogen percentage. We hypothesize that this range may represent the naturally occurring ranges of contamination in the production environment among the specific produce commodity, farm and packing facility, and type of pathogen. A second hypothesis may be that the range of pathogen prevalence analyzed may be due to the inherent variability of pooling data extracted from individual articles across regions, detection methods, and times.

When pooling pathogens, viruses and parasites exhibited higher prevalence on fresh produce than bacteria. One explanation for these results may stem from foodborne viruses’ (e.g., norovirus, hepatitis A, and rotavirus) increased ability to persist in the environment. A study examining the inactivation of caliciviruses reported the long-term survival (e.g., 1 week for 3 log_10_ reduction in infectivity at 20°C) at multiple, varying temperatures (60). In addition, a review of the survival of hepatitis A concluded that hepatitis A has a high half-life (e.g., 7.8 days at 20°C), regardless of the environmental humidity, and persists on both inanimate and animate surfaces (61). For parasites, research studying the survival rate of *Ascaris* eggs reported an inactivation rate of 180 days at 30°C and a pH of 7 (62). An additional hypothesis for the increased prevalence of viruses and parasites over bacteria results from the methodologies for the detection of the pathogens. For example, molecular-based tests for the detection of viruses generally have higher sensitivity than traditional culture-based tests for the detection of bacteria (63) and microscopy-based tests for the detection of parasites (64).

This research had several strengths and limitations. In particular, the systematic review approach employed a robust strategy to answer the research question on pathogen prevalence. The collection of primary data to address this question would have required a large investment of time and resources worldwide. The diversity of commodities, agroecologies, and seasons represented in the dataset enable conclusions relevant to many produce production environments. An additional strength of the present study included the methodology of the search for articles. The researchers validated the search string with articles intended for inclusion, and multiple databases were examined to increase the article yield. The inclusion of all commodities, pathogens, or countries limited the researchers’ biases and contributed to the sensitivity and comprehensiveness of the search. Further, two researchers independently performed each step of the data collection, which added to the rigor of the work. One limitation in the search and data extraction stage could include publication bias. Publication bias (a greater likelihood of publishing a positive, compared to a negative, pathogen prevalence) may have increased the bias of the extracted data toward positive outcomes. Interestingly, despite this possible publication bias, we observed low pathogen prevalence estimates. Additional limitations occurred in the analysis stage. For example, the data were pooled for analysis, which may have increased the variability and error in the estimates. The pooled data came from multiple pathogen detection assays across regions, methods, and times. In addition, concentration data reported as CFU or other measures of quantity of pathogens per positive sample was not included in the analyses. Due to the different laboratory detection methods and reporting units, the concentration data could not be accurately pooled, and compared. This variation in detection and reporting methods presents an opportunity for standardization of pathogen detection techniques on produce commodities, especially for the less common parasitic pathogens and viral pathogens that are un-culturable or are difficult to culture, like norovirus (3, 65–67). In addition, for the analysis, researchers assumed that stated percentage of pathogens detected represented the produce group sampled. However, the articles used different detection methodologies with varied limits of detection sensitivities and specificities, which limit the ability to detect true differences between groups. Future research that includes a larger dataset can address this issue.

Individuals involved in the fresh produce industry and food safety industry can use this review to identify locations and commodities with elevated contamination risk, and this information can contribute to ameliorate food safety practices. In addition, the low percentages of contamination on farms and packing facilities raise questions about its link with the frequency of produce-associated outbreaks. If a high frequency of produce-associated outbreaks is associated with a high percentage of pathogen contamination, then, most outbreaks probably do not originate from the farm and packing facility. Contamination of produce with pathogens may occur during other stages in the process between processing, handling, and consumption. Alternatively, a relationship between the frequency of produce-associated outbreaks and the frequency of pathogen contamination on produce may not exist. Instead, the outbreaks may result from the “perfect storm” of simultaneously occurring events (e.g., accidental pathogen contamination of fresh produce by a grower combined with conducive environmental conditions and protection of pathogens by the specific surface of a commodity (e.g., cantaloupe) and with packing practices that cross-contaminate pathogens). In addition, each pathogen strain has varying ranges of doses required to infect and sicken individuals and be propagated through person-to-person outbreaks. For example, certain strains of norovirus have an ID50 of 18 viruses (68), and thus, small amounts of virus on multiple individual produce commodities may be sufficient to cause a norovirus produce-associated outbreak.

Additional primary research would not only strengthen the results of this systematic review study but also contribute to answering questions about the relationships of outbreaks to pathogen prevalence on farms and packing facilities. Specifically, cross-sectional studies that follow the same produce commodity across the farm-to-fork continuum (upon arrival from the packing facilities and at the time of placement on the markets’ shelves) can quantify the percentage of pathogens at multiple stages. Further, additional research should continue to quantify the variability of pathogen contamination by commodity across multiple geographic locales and environmental conditions.

## Conclusion

Foodborne illness due to consumption of contaminated produce causes significant burden around the world. Results indicated a low median percentage of fresh produce contaminated with pathogens (0%).The quantification of the contamination of human pathogens on fresh produce on farms and in packing facilities improves our understanding of naturally occurring ranges of contamination in the production environment. Additional research to collect primary data in cross-sectional studies will strengthen the conclusions, but this review identifies contamination prevalence to inform strategies to prevent future produce-associated foodborne illness.

## Author Contributions

AV developed the study design, completed data analysis, and wrote and revised all drafts including the final manuscript. BQ developed the study design, completed data analysis, and reviewed drafts including the final manuscript. HL developed the study design, completed data analysis and final manuscript formatting, edits, and reviewed the final manuscript draft. FB conceived the idea, the study design, and reviewed the final manuscript draft. KN contributed to the study design and reviewed the final manuscript draft. JL conceived the idea, the study design, completed data analysis, and reviewed drafts including the final manuscript.

## Conflict of Interest Statement

The authors declare that the research was conducted in the absence of any commercial or financial relationships that could be construed as a potential conflict of interest.
