# Supplementary material for: Low Prevalence of Human Pathogens on Fresh Produce on Farms and in Packing Facilities: A Systematic Review
Source: Front Public Health. 2018 Feb 23;6:40. doi: 10.3389/fpubh.2018.00040 (PMC5829028; doi:10.3389/fpubh.2018.00040)
Supplement: Supplementary file 1 [file data_sheet_1.docx]

SUPPLEMENTARY TABLES

| TABLE 8  Pathogen Prevalence by Herb Commodity | | | | | | | | | | | | | | | | |
| --- | --- | --- | --- | --- | --- | --- | --- | --- | --- | --- | --- | --- | --- | --- | --- | --- |
|  | Bacteria | | | | | Parasites | | | | | Viruses | | | | | References |
| Commodity | Median Prevalence^ (Range) | Number of Groups Sampled^+^ | Number of Studies | Pathogens Tested | Sample Country | Median Prevalence^ (Range) | Number of Groups Sampled^+^ | Number of Studies | Pathogens Tested | Sample Country | Median Prevalence^ (Range) | Number of Groups Sampled^+^ | Number of Studies | Pathogens Tested | Sample Country |  |
| basil | - | - | - | - | - | 6.80  (1.1-12.5) | 2 | 2 | *Cryptosporidium, Cyclospora* | Iran, Vietnam | - | - | - | - | - | ([59](#_ENREF_59), [61](#_ENREF_61)) |
| cilantro | 0 (0) | 3 | 1 | *E. coli* O157:H7*, Listeria, Salmonella* | United States | - | - | - | *-* | - | 20.0 (0-40.0) | 3 | 1 | Hepatitis A, Norovirus, Rotavirus | Mexico | ([38](#_ENREF_38), [66](#_ENREF_66)) |
| coriander | - | - | - | - | - | 8.30  (6.70-14.2) | 2 | 2 | *Cryptosporidium, Cyclospora* | Iran, Vietnam | - | - | - | - | - | ([59](#_ENREF_59), [61](#_ENREF_61)) |
| cress | - | - | - | - | - | 8.90 | 1 | 1 | *Cryptosporidium* | Iran | - | - | - | - | - | ([59](#_ENREF_59)) |
| fennel | - | - | - | - | - | 0 (0) | 6 | 1 | *Ascaris* spp., *Toxocara* spp., *Trichuris* spp. | Poland | - | - | - | - | - | ([58](#_ENREF_58)) |
| dill | 0 | 5 | 3 | *E. coli* O157:H7*, Listeria,* Salmonella, Shiga toxin-producing *E. coli* | Greece, Poland, United States | - | - | - | *-* | - | - | - | - | - | - | ([38](#_ENREF_38), [41](#_ENREF_41), [44](#_ENREF_44)) |
| Indian pennywort | 0 (0) | 1 | 1 | *Campylobacter* | Malaysia | - | - | - | *-* | - | - | - | - | - | - | ([30](#_ENREF_30)) |
| marjoram | - | - | - | - | - | 16.6 | 1 | 1 | *Cyclospora* | Vietnam | - | - | - | - | - | ([61](#_ENREF_61)) |
| mint | - | - | - | - | - | 8.50 | 1 | 1 | *Cryptosporidium* | Iran | - | - | - | - | - | ([59](#_ENREF_59)) |
| parsley | 0 (0) | 5 | 3 | *E. coli* O157:H7*, Listeria, Salmonella,* Shiga toxin-producing *E. coli* | Greece, Lebanon, United States | 0 (0) | 6 | 1 | *Ascaris* spp., *Toxocara* spp., *Trichuris* spp. | Poland | 50.0 (0-50.0) | 3 | 1 | Hepatitis A, Norovirus, Rotavirus | Mexico | ([38](#_ENREF_38), [41](#_ENREF_41), [50](#_ENREF_50), [58](#_ENREF_58), [66](#_ENREF_66)) |
| sorrel | - | - | - | *-* | - | 0 (0) | 6 | 1 | *Ascaris* spp., *Toxocara* spp., *Trichuris* spp*.* | Poland | - | - | - | - | - | ([58](#_ENREF_58)) |
| Vietnamese coriander | 41.25 (12.5-70.0) | 2 | 2 | *Campylobacter* | Malaysia | - | - | - | *-* | - | - | - | - | - | - | ([30](#_ENREF_30), [31](#_ENREF_31)) |
| Vietnamese mint | - | - | - | - | - | 7.60 | 1 | 1 | *Cyclospora* | Vietnam | - | - | - | - | - | ([61](#_ENREF_61)) |
| ^Median % positive  ^+^Number of groups of commodities | | | | | | | | | | | | | | | |  |

| TABLE 9  Pathogen Prevalence by Fruit Commodity | | | | | | | | | | | | | | | | |
| --- | --- | --- | --- | --- | --- | --- | --- | --- | --- | --- | --- | --- | --- | --- | --- | --- |
|  | Bacteria | | | | | Parasites | | | | | Viruses | | | | | References |
| Commodity | Median Prevalence^ (Range) | Number of Groups Sampled^+^ | Number of Studies | Pathogens Tested | Sample Country | Median Prevalence^ (Range) | Number of Groups Sampled^+^ | Number of Studies | Pathogens Tested | Sample Country | Median Prevalence^ (Range) | Number of Groups Sampled^+^ | Number of Studies | Pathogens Tested | Sample Country |  |
| apple | 0 (0) | 4 | 1 | *E. coli* O157:H7*, Salmonella* | United States | - | - | - | - | - | - | - | - | - | - | ([37](#_ENREF_37)) |
| blackberry | 0 (0) | 1 | 1 | *Listeria* | Poland | - | - | - | - | - | - | - | - | - | - | ([45](#_ENREF_45)) |
| blueberry | 0 (0) | 1 | 1 | *Listeria* | Poland | - | - | - | - | - | - | - | - | - | - | ([45](#_ENREF_45)) |
| cantaloupe | 1.65 (0-40.0) | 4 | 2 | *E. coli* O157:H7*, Listeria, Salmonella* | Mexico, United States | - | - | - | - | - | - | - | - | - | - | ([38](#_ENREF_38), [52](#_ENREF_52)) |
| fruit^∆^ | 0 (0-4.00) | 6 | 3 | *E. coli* O157:H7, *Listeria, Salmonella* | Germany, South Africa, United States | - | - | - | - | - | - | - | - | - | - | ([35](#_ENREF_35), [46](#_ENREF_46), [56](#_ENREF_56)) |
| kiwifruit | 0.50 (0-3.90) | 6 | 1 | *Pathogenic E. coli, Staphylococcus aureus, Salmonella* | China | - | - | - | - | - | - | - | - | - | - | ([55](#_ENREF_55)) |
| orange | 0 (0) | 1 | 1 | *Salmonella* | United States | - | - | - | - | - | - | - | - | - | - | ([65](#_ENREF_65)) |
| peach | 2.50 (0-10.0) | 5 | 1 | *E. coli* O157:H7, *Listeria*, *Salmonella, Staphylococcus aureus* | South Africa | - | - | - | - | - | - | - | - | - | - | ([49](#_ENREF_49)) |
| raspberry | 0 (0-10.0) | 4 | 2 | *E. coli* O157:H7*, Listeria, Salmonella* | Multi-country*, Poland | - | - | - | - | - | 40.0 | 1 | 1 | Norovirus | Multi-country* | ([33](#_ENREF_33), [45](#_ENREF_45)) |
| Satsuma mandarin | 0 (0) | 2 | 1 | *Salmonella,* Verotoxin-producing *E. coli* | Japan | - | - | - | - | - | - | - | - | - | - | ([42](#_ENREF_42)) |
| strawberry | 0 (0-7.00) | 8 | 3 | *E. coli* O157:H7*, Listeria, Salmonella* | Poland, Spain, United States | 0 (0) | 6 | 1 | *Ascaris* spp., *Toxocara* spp., *Trichuris* spp. | Poland | 30.0 | 1 | 1 | Norovirus | Spain | ([33](#_ENREF_33), [37](#_ENREF_37), [45](#_ENREF_45), [58](#_ENREF_58)) |
| tangerine | 0 (0) | 1 | 1 | *Salmonella* | United States | - | - | - | *-* | - | - | - | - | - | - | ([65](#_ENREF_65)) |
| ^Median % positive  ^+^Number of groups of commodities  ^∆^specific commodities not articulated  *Data aggregated for more than one country | | | | | | | | | | | | | | | | |

| TABLE 10  Pathogen Prevalence by Vegetable Commodity | | | | | | | | | | | | | | | | |
| --- | --- | --- | --- | --- | --- | --- | --- | --- | --- | --- | --- | --- | --- | --- | --- | --- |
|  | Bacteria | | | | | Parasites | | | | | Viruses | | | | | References |
| Commodity | Median Prevalence^ (Range) | Number of Groups Sampled^+^ | Number of Studies | Pathogens Tested | Sample Country | Median Prevalence^ (Range) | Number of Groups Sampled^+^ | Number of Studies | Pathogens Tested | Sample Country | Median Prevalence^ (Range) | Number of Groups Sampled^+^ | Number of Studies | Pathogens Tested | Sample Country |  |
| arugula | 0 | 3 | 1 | *E. coli* O157:H7, *Listeria* | United States | - | - | - | - | - | - | - | - | - | - | ([38](#_ENREF_38)) |
| baby spinach | 0 (0-5.60) | 8 | 2 | *E. coli* O157:H7, *Listeria, Salmonella* | South Africa, Spain | - | - | - | - | - | - | - | - | - | - | ([32](#_ENREF_32), [68](#_ENREF_68)) |
| beetroot | 0 (0) | 2 | 1 | *Salmonella,* Shiga toxin- producing *E. coli* | Greece, Poland | 16.7 (0-16.7) | 6 | 1 | *Ascaris* spp., Toxocara spp., *Trichuris* spp. | Poland | - | - | - | - | - | ([41](#_ENREF_41), [44](#_ENREF_44), [58](#_ENREF_58)) |
| bok choi | 0 (0) | 4 | 1 | *E. coli* O157:H7, *Salmonella,* | United States | - | - | - | *-* | - | - | - | - | - | - | ([37](#_ENREF_37)) |
| brinjal | 20.0 | 1 | 1 | *Listeria* | India | - | - | - | *-* | - | - | - | - | - | - | ([48](#_ENREF_48)) |
| broccoli | 0 (0) | 5 | 2 | *E. coli* O157:H7, *Listeria, Salmonella* | India, United States | 0 (0) | 6 | 1 | *Ascaris* spp., *Toxocara* spp., *Trichuris* spp. | Poland | - | - | - | - | - | ([37](#_ENREF_37), [48](#_ENREF_48), [58](#_ENREF_58)) |
| bulbous vegetables^∆^ | 0.85 (0-1.70) | 2 | 1 | *Listeria, Salmonella* | Germany | - | - | - | *-* | - | - | - | - | - | - | ([46](#_ENREF_46)) |
| cabbage | 0 (0-50.0) | 15 | 8 | *Campylobacter, E. coli* O157:H7, *Listeria, Salmonella, Shigella* | Egypt, Eritrea, India, Malaysia, Poland, South Africa, United States | 0 (0-50.0 | 7 | 2 | *Ascaris* spp., *Giardia, Toxocara* spp., *Trichuris* spp. | Eritrea, Poland | 0 (0) | 3 | 1 | Hepatitis A, Norovirus, Rotavirus | Mexico | ([30](#_ENREF_30), [37](#_ENREF_37), [45](#_ENREF_45), [47](#_ENREF_47), [48](#_ENREF_48), [54](#_ENREF_54), [57](#_ENREF_57), [58](#_ENREF_58), [66](#_ENREF_66), [68](#_ENREF_68)) |
| carrot | 0 (0-4.00) | 8 | 4 | *Salmonella,* Shiga toxin-producing *E. coli, Shigella* | Eritrea, Greece, United States | 0 (0-42.9) | 7 | 2 | *Ascaris* spp., *Giardia, Toxocara* spp., *Trichuris* spp. | Eritrea, Poland | - | - | - | - | - | ([41](#_ENREF_41), [44](#_ENREF_44), [53](#_ENREF_53), [54](#_ENREF_54), [58](#_ENREF_58)) |
| cauliflower | 20.0 | 1 | 1 | *Listeria* | India | 0 (0) | 6 | 1 | *Ascaris* spp., *Toxocara* spp., *Trichuris* spp. | Poland | - | - | - | - | - | ([48](#_ENREF_48), [58](#_ENREF_58)) |
| celery | 0 (0) | 1 | 1 | Shiga toxin producing *E. coli* | Greece | 0 (0-25) | 7 | 2 | *Ascaris* spp., *Cyclospora, Toxocara* spp., *Trichuris* spp. | Italy, Poland | - | - | - | - | - | ([41](#_ENREF_41), [58](#_ENREF_58), [62](#_ENREF_62)) |
| chappan-kaddu | 10.0 | 1 | 1 | *Listeria* | India | - | - | - | *-* | - | - | - | - | - | - | ([48](#_ENREF_48)) |
| cherry tomato | 0 (0) | 3 | 1 | *E. coli* O157:H7*, Listeria, Salmonella* | Spain | - | - | - | - | - | 23.3 | 1 | 1 | Norovirus | Spain | ([33](#_ENREF_33)) |
| chile pepper | 60.0 | 1 | 1 | *Salmonella* | Mexico | - | - | - | *-* | - | - | - | - | - | - | ([52](#_ENREF_52)) |
| chili | 10.0 | 1 | 1 | *Listeria* | India | - | - | - | *-* | - | - | - | - | - | - | ([48](#_ENREF_48)) |
| collards | 0 | 3 | 1 | *E. coli* O157:H7, *Listeria, Salmonella* | United States | - | - | - | - | - | - | - | - | - | - | ([38](#_ENREF_38)) |
| cowpea | 0 (0) | 1 | 1 | *Listeria* | India | - | - | - | *-* | - | - | - | - | - | - | ([48](#_ENREF_48)) |
| cucumber | 0 (0-70.0) | 6 | 2 | *Campylobacter, E. coli* O157:H7, *Salmonella* | Malaysia, United States | 9.10 | 1 | 1 | *Cyclospora* | Italy | - | - | - | - | - | ([31](#_ENREF_31), [37](#_ENREF_37), [62](#_ENREF_62)) |
| cucurbits | 0 (0) | 2 | 1 | *Shigella, Salmonella* | Eritrea | 0 (0) | 1 | 1 | *Giardia* | Eritrea | - | - | - | - | - | ([54](#_ENREF_54)) |
| dolichos bean | 20.0 | 1 | 1 | *Listeria* | India | - | - | - | *-* | - | - | - | - | - | - | ([48](#_ENREF_48)) |
| French beans | - | - | - | *-* | - | 0 (0) | 6 | 1 | *Ascaris* spp., *Trichuris* spp., *Toxocara* spp. | Poland | - | - | - | - | - | ([58](#_ENREF_58)) |
| garlic | 0 (0) | 1 | 1 | Shiga toxin- producing *E. coli* | Greece | - | - | - | *-* | - | - | - | - | - | - | ([41](#_ENREF_41)) |
| green onion | - | - | - | *-* | - | 14.8 | 1 | 1 | *Cryptosporidium* | Iran | 22.2 (11.1-22.2) | 3 | 1 | Hepatitis A, Norovirus, Rotavirus | Mexico | ([59](#_ENREF_59), [66](#_ENREF_66)) |
| green pepper | 0 (0-2.32) | 4 | 1 | *E. coli* O157:H7, *Salmonella* | United States | - | - | - | *-* | - | - | - | - | - | - | ([37](#_ENREF_37)) |
| jalapeño pepper | - | - | - | *-* | - | - | - | - | *-* | - | 0 | 3 | 1 | Hepatitis A, Norovirus, Rotavirus | Mexico | ([66](#_ENREF_66)) |
| kale | 0 (0) | 2 | 1 | *Salmonella* | Kenya | - | - | - | *-* | - | - | - | - | - | - | ([51](#_ENREF_51)) |
| leafy greens | 0 (0) | 6 | 2 | *E. coli* O157:H7*, Salmonella, Shigella* | Eritrea, United States | 25.0 | 1 | 1 | *Giardia* | Eritrea | - | - | - | - | - | ([37](#_ENREF_37), [54](#_ENREF_54)) |
| leek | 0 (0) | 1 | 1 | Shiga toxin- producing *E. coli* | Greece | 0 (0-8.33) | 7 | 2 | *Ascaria* spp., *Toxocara* spp., *Trichuris* spp., *Cryptosporidium* | Iran, Poland | - | - | - | - | - | ([41](#_ENREF_41), [58](#_ENREF_58), [59](#_ENREF_59)) |
| lettuce | 0 (0-20.0) | 19 | 15 | *Campylobacter, E. coli* O157:H7, *Enterohaemorrhagic E. coli, Hafnia alvei, Listeria, Salmonella,* Shiga toxin-producing *E. coli, Shigella* | Belgium, Brazil, Egypt, Eritrea, Greece, Lebanon, Netherlands, Poland, South Africa, United States | 0 (0-50.0) | 8 | 2 | *Ascaris* spp., *Cyclospora, Toxocara* spp., *Trichuris* spp. | Poland, Vietnam | 0 (0) | 3 | 1 | Hepatitis A, Norovirus, Rotavirus | Mexico | ([36](#_ENREF_36), [37](#_ENREF_37), [39](#_ENREF_39), [41](#_ENREF_41), [43-45](#_ENREF_43), [50](#_ENREF_50), [54](#_ENREF_54), [57](#_ENREF_57), [58](#_ENREF_58), [61](#_ENREF_61), [66](#_ENREF_66), [68](#_ENREF_68)) |
| long yard bean | 25.0 (0-50.0) | 2 | 2 | *Campylobacter* | Malaysia | - | - | - | *-* | - | - | - | - | - | - | ([30](#_ENREF_30), [31](#_ENREF_31)) |
| mustard greens | 0 | 3 | 1 | *E. coli* O157:H7, *Listeria, Salmonella* | United States | - | - | - | *-* | - | - | - | - | - | - | ([38](#_ENREF_38)) |
| onion | 0 (0) | 7 | 2 | *E. coli* O157:H7, *Listeria, Salmonella* | South Africa, United States | 0 (0-28.6) | 7 | 2 | *Ascaris* spp., *Trichuris* spp., *Toxocara* spp. | Poland, South Africa | - | - | - | - | - | ([34](#_ENREF_34), [37](#_ENREF_37), [41](#_ENREF_41), [58](#_ENREF_58)) |
| palak | 0 (0) | 1 | 1 | *Listeria* | India | - | - | - | *-* | - | - | - | - | - | - | ([48](#_ENREF_48)) |
| potato | - | - | - | *-* | - | 0 (0-33.3) | 6 | 1 | *Ascaris* spp., *Toxocara* spp., *Trichuris* spp. | Poland | - | - | - | - | - | ([58](#_ENREF_58)) |
| pumpkin | - | - | - | *-* | - | 0(0) | 6 | 1 | *Ascaris* sp., *Toxocara* spp., *Trichuris* spp*.* | Poland | - | - | - | - | - | ([58](#_ENREF_58)) |
| radish | 0 (0-16.7) | 3 | 3 | *Campylobacter, Salmonella* | Lebanon, Malaysia, Poland | - | - | - | *-* | - | - | - | - | - | - | ([30](#_ENREF_30), [44](#_ENREF_44), [50](#_ENREF_50)) |
| rhubarb | - | - | - | *-* | - | 33.3 (0-33.3) | 6 | 1 | *Ascaris* spp., *Toxocara* spp., *Trichuris* spp. | Poland | - | - | - | - | - | ([58](#_ENREF_58)) |
| Romaine lettuce | 3.64 (0-7.93) | 10 | 1 | *Bacillus cereus, E. coli* O157:H7, *Listeria, Salmonella,*  *Staphylococcus aureus* | Korea | - | - | - | - | - | - | - | - | - | - | ([29](#_ENREF_29)) |
| Roman rocket | 0 (0) | 1 | 1 | Shiga toxin- producing *E. coli* | Greece | - | - | - | - | - | - | - | - | - | - | ([41](#_ENREF_41)) |
| root vegetables^∆^ | 1.50 (0-3.00) | 2 | 1 | *Listeria, Salmonella* | Germany | - | - | - | - | - | - | - | - | - | - | ([46](#_ENREF_46)) |
| salad | 0.80 (0-1.60) | 2 | 1 | *Listeria, Salmonella* | Germany | - | - | - | - | - | - | - | - | - | - | ([46](#_ENREF_46)) |
| sesame leaf | 0 (0-7.93) | 10 | 1 | *Bacillus cereus, E. coli* O157:H7, *Listeria, Salmonella,*  *Staphylococcus aureus* | Korea | - | - | - | - | - | - | - | - | - | - | ([29](#_ENREF_29)) |
| spinach | 0 (0-11.1) | 16 | 4 | *Bacillus cereus, E. coli* O157:H7, *Listeria, Salmonella,* Shiga toxin-producing *E. coli, Shigella, Staphylococcus aureus* | Greece, Korea, Multi-country, United States | - | - | - | - | - | - | - | - | - | - | ([29](#_ENREF_29), [38](#_ENREF_38), [41](#_ENREF_41), [63](#_ENREF_63)) |
| summer squash | 0 (0) | 4 | 1 | *E. coli* O157:H7, *Salmonella* | United States | - | - | - | - | - | - | - | - | - | - | ([37](#_ENREF_37)) |
| tomato | 0 (0) | 11 | 5 | *Campylobacter, E. coli* O157:H7, *Listeria, Salmonella,* Shiga toxin-producing *E. coli, Shigella* | Eritrea, Malaysia, Poland, United States | 22.2 | 1 | 1 | *Giardia* | Eritrea | - | - | - | - | - | ([30](#_ENREF_30), [37](#_ENREF_37), [40](#_ENREF_40), [45](#_ENREF_45), [48](#_ENREF_48), [54](#_ENREF_54)) |
| turnip | - | - | - | *-* | - | 0 (0) | 6 | 1 | *Ascaris* spp., *Toxocara* spp., *Trichuris* spp. | Poland | - | - | - | - | - | ([58](#_ENREF_58)) |
| vegetables^∆^ | 0 (0) | 2 | 1 | *E. coli* O157:H7, *Salmonella* | United States | - | - | - | *-* | - | - | - | - | - | - | ([35](#_ENREF_35)) |
| water spinach | 10.0 (0-20.0) | 2 | 2 | *Campylobacter* | Malaysia | 17.0 (0-100) | 25 | 1 | *Cryptosporidium, Cyclospora, Giardia,* Helminth eggs | Cambodia | - | - | - | - | - | ([31](#_ENREF_31), [60](#_ENREF_60)) |
| wild cosmos | 65.0 (30.0-90.0) | 4 | 1 | *Campylobacter* | Malaysia | - | - | - | *-* | - | - | - | - | - | - | ([31](#_ENREF_31)) |
| winged bean | 6.67 | 1 | 1 | *Campylobacter* | Malaysia | - | - | - | *-* | - | - | - | - | - | - | ([31](#_ENREF_31)) |
| young beetroot leaves | - | - | - | *-* | - | 0 (0-100) | 6 | 1 | *Ascaris* spp., *Toxocara* spp., *Trichuris* spp. | Poland | - | - | - | - | - | ([58](#_ENREF_58)) |
| zucchini | 0 (0) | 4 | 1 | *E. coli* O157:H7, *Salmonella* | United States | 0 (0-14.3) | 6 | 1 | *Ascaris* spp., *Toxocara* spp., *Trichuris* spp. | Poland | - | - | - | - | - | ([37](#_ENREF_37), [58](#_ENREF_58)) |
| ^Median % positive  ^+^Number of groups of commodities  ^∆^specific commodities not articulated | | | | | | | | | | | | | | | | |
